# *Crataeva nurvala* Bark (Capparidaceae) Extract Modulates Oxidative Stress-Related Gene Expression, Restores Antioxidant Enzymes, and Prevents Oxidative Stress in the Kidney and Heart of 2K1C Rats

**DOI:** 10.1155/2023/4720727

**Published:** 2023-08-09

**Authors:** Ishrat Jahan, Proma Saha, Tammana Tabassum Eysha Chisty, Kaniz Fatima Mitu, Faizul Islam Chowdhury, Khondoker Shahin Ahmed, Hemayet Hossain, Ferdous Khan, Nusrat Subhan, Md. Ashraful Alam

**Affiliations:** ^1^Department of Pharmaceutical Sciences, North South University, Dhaka, Bangladesh; ^2^Chemical Research Division, BCSIR Laboratories, Dhaka, Bangladesh Council of Scientific and Industrial Research (BCSIR), Dhaka-1205, Bangladesh; ^3^Institute of Food Science and Technology (IFST), Bangladesh Council of Scientific and Industrial Research (BCSIR), Dhaka 1205, Bangladesh

## Abstract

**Objective:**

*Crataeva nurvala* is a medicinal plant, which contains a wide range of polyphenolic and bioactive compounds. The aim of the study was to evaluate the renal-protective activity of *Crataeva nurvala* in two-kidney, one-clip (2K1C) rats.

**Methods:**

In this study, the ethanol extract of *Crataeva nurvala* bark at a dose of 100 mg/kg was orally used to treat 2K1C rats for four weeks. At the end of the experiment, all rats were sacrificed and tissue samples were collected for further biochemical and histological assessments.

**Results:**

This investigation showed that *Crataeva nurvala* treatment prevented the kidney dysfunction in 2K1C rats. Uric acid and creatinine concentration and CK-MB activities increased in 2K1C rats which were normalized by *Crataeva nurvala*. 2K1C rats also showed increased oxidative stress, depicted by the elevated level of MDA, NO, and APOP in plasma and tissues. Oxidative stress parameters declined in 2K1C rats by the treatment of *Crataeva nurvala*. These results could be attributed to the restoration of antioxidant enzyme activities such as catalase and SOD. *Crataeva nurvala* extracts also upregulated antioxidant gene expression in the kidneys of 2K1C rats. Moreover, several anti-inflammatory genes were suppressed by *Crataeva nurvala* treatment in 2K1C rats. Furthermore, fibrosis and collagen deposition in the kidneys were also lowered by the treatment of the *Crataeva nurvala* extract.

**Conclusion:**

The experimental data suggest that the *Crataeva nurvala* extract protected renal damage and oxidative stress, probably by restoring antioxidant enzymes activities in 2K1C rats.

## 1. Introduction

Kidney dysfunction has emerged as a rising problem worldwide in the last few years [1, 2]. It has become a significant risk factor for all mortality, including cardiovascular disease (CVD) events around the world [3]. The kidneys have various important regulatory functions in the human body, including purifying body fluid and regulating blood pressure [4]. Chronic kidney disease signals the development of hypertension, the world's most common cause of end-stage renal failure at the moment, not just in developed regions but also in developing nations [5, 6]. Patients with renal failure, who need replacement therapy, have a rising incidence and prevalence but have poor outcomes and substantial expenditures [7]. In the case of chronic kidney disease and hypertension, renin-angiotensin-aldosterone system (RAAS) activation may play a vital role in the development of these diseases [8]. An ideal model to investigate RAS-mediated complications is the two-kidney, one-clip (2K1C) model in rats [9, 10]. An excess production of angiotensin II along with aldosterone secretion is considered an overactivated RASS system, which may lead to renal dysfunction followed by an increase in glomerular pressure, facilitating glomerular arteriosclerosis. In addition, reactive oxygen species (ROS) leading to increased oxidative stress, activation of nuclear factor kappa B (NF-*κ*B), and release of cytokines and chemokines are all mediated through increased Ang-II, eventually leading to renal glomerular sclerosis and injury [11–13].

Reactive nitrogen species (RNS) and reactive oxygen species (ROS) may be produced by cellular metabolism [14]. However, endogenous antioxidants function as part of a comprehensive system that incorporates its main constituents in order to maintain redox balance in our body, where main enzymes such as catalase (CAT), superoxide dismutase (SOD), and glutathione peroxidase (GPx) eradicate ROS/RNS and shield cells from their harmful effects [15–17]. Moreover, ROS/RNS induce cellular injury and inflammation when their synthesis outpaces the antioxidant system's ability to neutralize them, which results in oxidative stress. According to the research, a number of chronic pathologies such as chronic kidney diseases and cardiovascular diseases have been closely associated with oxidative stress and subsequent inflammatory responses [15].

Antioxidants have been found to be helpful in preventing or reversing oxidative cell damage because of their free radical scavenging abilities. Evidence suggests that polyphenolic antioxidants are present in fruits, vegetables, beverages, and various herbs. These antioxidants may prevent oxidative stress and reduce the progression of ischemic heart disease, atherosclerosis, hypertension, stroke, diabetes, and chronic kidney diseases [18, 19]. Thus, natural antioxidants are considered useful due to their ability to scavenge ROS, which may prevent chronic kidney disease [19].

*Crataeva nurvala* has been described in much remarkable qualitative research to have numerous therapeutic activities and is widely used in the field of many Ayurvedic and Unani medicine formularies as well [20]. An earlier literature report showed that this plant has antiarthritic, hepatoprotective, and cardioprotective actions [21]. In addition, this medicinal plant relieves, prevents, and promotes the discharge of kidney stones [21]. Different parts of *Crataeva nurvala* such as leaves, barks, and roots are important components in terms of therapeutic effects [20]. Numerous health benefits of *Crataeva nurvala* have been reported including protection against oxidative stress [20], prevention and treatment of convulsions [22], gastric irritation [23], cancer [24], obesity, thyroid disorder, and urinary stone [25]. Barks of *Crataeva nurvala* as well as its leaves and roots have been reported to contain antioxidant compounds such as flavonoids (L-stachydrine, rutin, quercetin-3-O-*α*-D-glucoside, quercetin, isoquercetin, methyl pentacosanoate, kaempferol-O-*α*-D-glucoside, and dodecanoic anhydride) and phenolics (linoleic acid, oleic acid, stearic acid, lauric acid, rutin, quercetin, *γ*-taraxasterol, lupeol, *β*-sitosterol acetate, *β*-sitosterol, and *β*-epilupeol) [26]. However, the effect of *Crataeva nurvala* on experimental kidney dysfunction has not been tested in any reports so far. Thus, in this present investigation, the pharmacological evaluation of the *Crataeva nurvala* bark extract was carried out for its *in vivo* antioxidant, anti-inflammatory, and nephroprotective activities in the 2K1C rat model.

## 2. Materials and Methods

### 2.1. Reagents and Chemicals

*p*-Coumaric acid, catechol, myricetin, caffeic acid, trans-cinnamic acid, (−) epicatechin, syringic acid, trans-ferulic acid, quercetin, 3,4-dihydroxybenzoic acid, rosmarinic acid, vanillic acid, gallic acid, kaempferol, and catechin hydrate were purchased from Sigma-Aldrich (St. Louis, MO, USA). Acetic acid (HPLC), ethanol, methanol (HPLC), and acetonitrile (HPLC) were obtained from Merck (Darmstadt, Germany). Aspartate transaminase (AST) (Ref. no. 30243), alanine transaminase (ALT) (Ref. no. 30253), and alkaline phosphatase (ALP) (Ref. no. 41257) kits were purchased from DCI Diagnostics (Budapest, Hungary). Creatinine kinase-muscle brain (CK-MB) (Ref. no. 41273), uric acid (Ref. no. 30393), and creatinine (Ref. no. 41262) kits were also obtained from DCI Diagnostics (Budapest, Hungary). GeneJET RNA Purification Kit (Catalog number: K0732), RevertAid First Strand cDNA Synthesis Kit (Catalog number: K1621), and SYBR™ Green PCR Master Mix (Catalog number: 4309155) were purchased from Thermo Fisher Scientific Inc. (Waltham, Massachusetts, United State of America).

### 2.2. Preparation of the Plant and Extract Preparation Procedure

The bark of plant *Cratavae nurvala* was collected from local markets of Mymensingh, Bangladesh. The plant was first identified/authenticated from National Herbarium, Mirpur, Bangladesh. For future reference, an accession number (DACB-78798) was deposited. The bark was cut into small pieces. After that, all bark pieces were ground into powder. Then, 200 gram powder was soaked in 80% ethanol for 7 days. The solvent was then decanted and filtered with filter paper. The extract was concentrated in a rotary evaporator at 40°C. A sticky crude extract was obtained. The percentage yield was calculated. It was then ready for further animal experiments and phytochemical screening. The eventual percentage yield of the plant extract from the soaked powder was 33.25 (w/w).

### 2.3. Working Standard Solution Preparation for HPLC Analysis

At first, standard stock solutions were prepared using 16 phenolic compounds by dissolving all of them separately in methanol in a 25 mL volumetric flask. The standard concentrations such as 4–50 *μ*g/mL were prepared from the stock solutions by a serial dilution method.

### 2.4. HPLC Analysis of *Crataeva nurvala* Bark Extract

To perform HPLC-DAD analysis of the *Crataeva nurvala* extract, a previous method with some modifications was used [27]. The HPLC instruments were used which have LC solution software (Lab Solution), LC-20A (Shimadzu, Japan) which was connected with a binary solvent delivery pump (LC-20AT), a column oven (CTO-20A), an autosampler (SIL-20A HT), and a photodiode array detector (SPD-M20A). At 33°C, a Luna C_18_ (5 *μ*m) Phenomenex column (4.6 × 250 mm) was used for the separation. Two mobile phases were involved such as A (1% acetic acid in acetonitrile) and B (1% acetic acid in water) with gradient elution: 0.01–20 min (5–25% A), 20–30 min (25–40% A), 30–35 min (40–60% A), 35–40 min (60−30% A), 40–45 min (30−5% A), and 45–50 min (5% A) were used in this study. The flow rate was fixed for 0.5 mL/min, whereas the volume of sample injection was 20 microliter. At 270 nm, a UV detector was set. Before starting the analysis procedure, a membrane filter of 0.45 micrometer was used to filter the mobile phases, and in addition, they were also degassed with the help of vacuum. Standard stock solutions were prepared to create a calibration curve. The standard stock compounds were gallic acid (20 *μ*g/ml), 3,4-dihydroxybenzoic acid (15 *μ*g/ml), catechin hydrate (50 *μ*g/ml), catechol, (−) epicatechin, caffeic acid, vanillic acid, syringic acid, rutin hydrate, *p*-coumaric acid, trans-ferulic acid, rosmarinic acid (30 *μ*g/ml each), myricetin, quercetin (10 *μ*g/ml each), trans-cinnamic acid (4 *μ*g/ml), and kaempferol (8 *μ*g/ml each).

### 2.5. Animals and Experimental Design

All animals used in this study were Long–Evans male rats (age 10–12 weeks and body weight 200–220 g). Twenty-four rats were taken from the Animal House of North South University, Bangladesh. Dark and light cycles (12 hours) were maintained, and every rat was kept in separate cages (room temperature 25 ± 2°C and humidity 45%). All rats were given access to food and water, and all procedures were approved by the IACUC (IACUC-No. 0907) of North South University, Bangladesh.

### 2.6. 2K1C Surgical Procedure

A surgical procedure was performed to develop the 2K1C rats, where the kidney artery was clipped with a plastic tube (2 mm long and 0.4 mm internal diameter) using the butterfly needle tube [28, 29]. The animals were anesthetized with 50 mg/kg ketamine (i.p.). The hair on the skin was shaved carefully, and a 2-3 cm long incision was made on the left side beside the spine. The left kidney was carefully taken out, and the adipose tissues were separated. For clipping, the renal artery of the left kidney was separated from the renal ureter. The plastic clip was placed around the renal artery carefully and tightened with the help of a suture knot, resulting in the partial occlusion of renal perfusion. The kidney was then gently pushed back into the retroperitoneal cavity. Blood was cleared with povidone-iodine, and the incision was closed layer by layer with sutures. Lidocaine gel was applied to the wound. In control rats, a sham surgical procedure was performed without clipping the artery. At the end of the experiment, this kidney became small and shrank significantly compared to the right kidney.

## 3. Animal Groupings

The rats were divided into four groups having 6 rats in each group.

The control group received only normal chaw food and water.

The rats in the 2K1C group underwent surgery, and the renal artery of the left kidney of the rats was clipped. They were also provided with 1% sodium chloride water with powdered chaw food.

The rats in the 2K1C + CN100 group were also treated same as those in the 2K1C group; in addition, they were administered with the *Crataeva nurvala* bark extract at a dose of 100 mg/kg/day orally for 4 weeks.

The dose of the *Crataeva nurvala* bark extract was selected based on a previous report found in a literature search [30].

The rats in the 2K1C + Ram group were also treated same as those in the 2K1C group; in addition, they were administered with ramipril at a dose of 10 mg/kg/day orally for 4 weeks.

### 3.1. Animal Sacrifice and Tissue Sample Preparation

When the experimental period was over, all animals were prepared for sacrifice. For euthanasia, high-dose pentobarbital (90 mg/kg) was used. Blood was collected using a 5 mL syringe with a 21 gauge needle. Citrate buffer (*pH* 7.4) was used as an anticlotting agent. Plasma was separated with centrifugation at 1,600 g at 4°C for 15 minutes. The plasma was transferred into a 1.5 mL microcentrifuge tube and kept at −20°C in a freezer for further study. The kidney and the heart were also collected immediately after the sacrifice of the animals. They were preserved into two parts: one part was for a biochemical assay and the other part was for histological staining. For the biochemical assay, organs were kept at −20°C. The other parts of the organs were kept in neutral buffered formalin (*pH* 7.4) for histological staining. The kidney and heart tissues were homogenized in phosphate buffer saline (PBS, *pH* 7.4) which was then centrifuged at 5,000 g at 4°C for 15 minutes. The supernatant solutions were collected and used for the biochemical assay.

### 3.2. Evaluation of Hepatic, Renal, and Cardiac Diagnostic Markers (ALT, AST, ALP, Uric acid, Creatinine, and CK-MB) in Plasma

Plasma samples were used to evaluate the ALT, AST, and ALP activities, uric acid and creatinine concentration, and CK-MB activity, using the assay kits followed by the manufacturer's protocol DCI diagnostics (Budapest, Hungary).

### 3.3. Oxidative Stress Markers (MDA, NO, and APOP) Assessment

For the measurement of lipid peroxidation, a colorimetric method was followed which was described previously [28]. For absorbance, the wavelength used was 532 nm.

To determine the nitric oxide level, a standard curve of nitrate was used, and NO was estimated following a procedure that was also described previously in the literature [28]. The units of NO were nmol/mL or nmol/g tissue.

Another test protocol was used to assay APOP, and the protocol was also previously described in the published literature [28].

### 3.4. Antioxidant Enzymes (Catalase and SOD Activities) Determination

The catalase activity assay was developed based on the hydrogen peroxide decay in the assay system and was described in a previous report [31]. The catalase activity unit was expressed as units/min.

The SOD activity assay used was described in the previously published literature [32]. In the sample solution, autooxidation of adrenaline was measured which was expressed as unit/mg. One unit enzyme activity is related to 50% reduction of the assay reaction.

### 3.5. Estimation of MPO Activity in Kidney and Heart Tissues

MPO is known as an inflammation marker in tissues. The MPO activity was measured using an *o*-dianisidine-H_2_O_2_ method described previously [33]. The absorbances were taken at 460 nm, and the unit of the MPO activity was expressed as MPO/mg protein.

### 3.6. RT-PCR for Oxidative Stress and Inflammation Regulatory Genes

Total 30 *μ*m RNA was isolated from the renal cortex using a Thermo Fisher Scientific's RNA purification kit (Massachusetts, USA). Following RNA quantification with NanoDrop 2000 (Bio-Rad, California, USA), 1 *μ*m of RNA from each sample was used to create cDNA using RevertAid First Strand cDNA Synthesis Kit according to the manufacturer's instructions (Thermo Fisher Scientific, USA). Primer 3 online software was used to design target gene primers (Table 1). The transcript levels of numerous components and enzymes were quantified using the Maxima SYBR Green qPCR master mix (Thermo Scientific, USA). PCR was carried out using a CFX96 C1000 Touch Real-Time PCR detection system (Bio-Rad, California, USA) in accordance with the previously established technique. The data analysis was performed CFX ManagerTM software (Bio-Rad, California, USA).

### 3.7. Histopathological Study

For a histological study, both kidney and heart tissues were kept in neutral buffered formalin for fixation. The fixed tissues underwent graded xylene treatment for tissue preparation and embedding into a paraffin block. They were then used for sectioning at 5 micrometer thickness using a rotary microtome. Hematoxylin and eosin (H&E) and sirius red staining was performed on the sectioned tissue samples. H&E staining was performed to check the normal structure of tissues [9]. Sirius red staining was performed for the collagen deposition in the kidney and heart [34]. The pictures were taken at 40x magnifications under a light microscope (Zeiss Axioscope).

The histological scoring system on kidney damage in lab animals was also used based on the EGTI scoring system, in which 4 separate components such as *endothelial, glomerular, tubular*, and *interstitial* damage in kidney sections were examined [35, 36]. The percentages of fibrosis were also determined in kidney and heart sections using Image*J* free software (version 4.0) from the National Institute of Health of United State of America [28, 37].

### 3.8. Statistical Analysis

The collected data were processed using appropriate statistical analysis. All data were presented as the mean ± standard error of means (SEM). All results from the four groups used in this study were analyzed by one-way ANOVA followed by a Tukey test. GraphPad prism software, USA (version 6.0) was used to analyze all the data. Statistical significance was considered as *P* < 0.05 in all cases, otherwise, specified in the table or figure.

## 4. Results

### 4.1. HPLC-DAD Analysis

**Figure 1:**
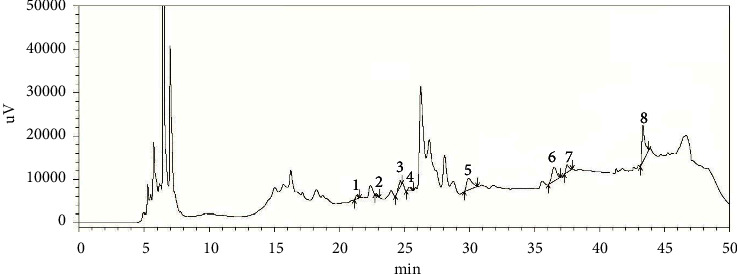
HPLC chromatograph of the *Crataeva nurvala* bark ethanol extract. Peaks: (1) catechin hydrate, (2) catechol, (3) (−) epicatechin, (4) vanillic acid, (5) rutin hydrate, (6) rosmarinic acid, (7) myricetin, and (8) trans-cinnamic acid.

To assess the presence of phenolic antioxidants in the sample, the HPLC method was used which simultaneously analyzed sixteen polyphenolic chemicals, and linearity, accuracy, stability, and precision were confirmed for the analysis [38]. In this experiment, the HPLC-DAD analytical method allowed the accurate identification and quantification of various phenolic components present in the *Crataeva nurvala* bark extract.  Figure 1 depicts the chromatographic separation of various polyphenols found in the *Crataeva nurvala* bark extract. As stated in Table 2, the quantity of each phenolic component identified in the *Crataeva nurvala* bark extract was determined using the matching calibration curve. Catechin hydrate, catechol, epicatechin, vanillic acid, rutin hydrate, rosmarinic acid, myricetin, and trans-cinnamic acid were found in the *Crataeva nurvala* bark extract.

### 4.2. Effect of the *Crataeva nurvala* Bark on and Organ Weights of 2K1C Rats

**Figure 2:**
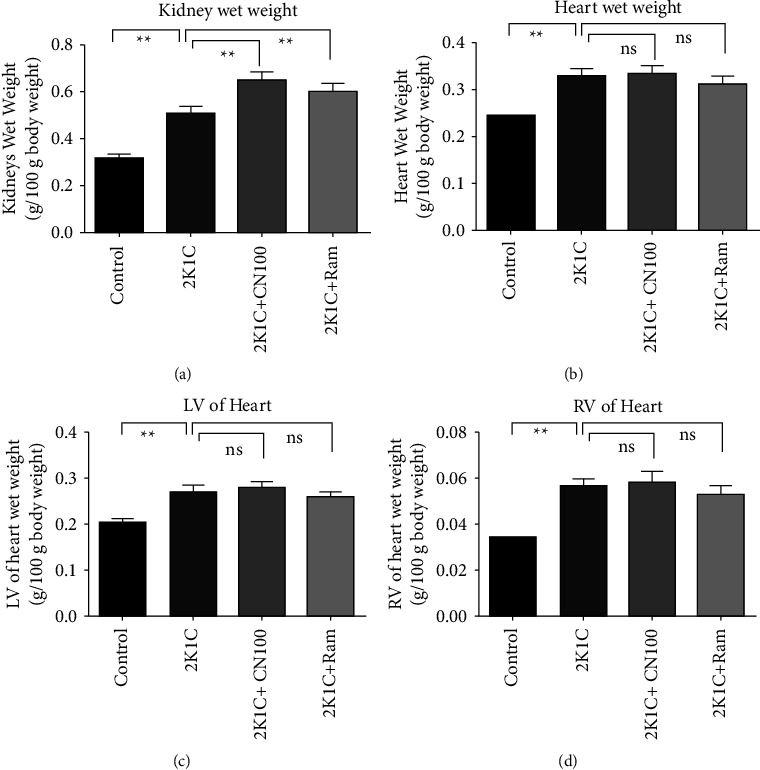
Effect of the *Crataeva nurvala* bark extract on kidney wet weight and heart wet weight of 2K1C rats. (a) Kidneys wet weight, (b) heart wet weight, (c) left ventricular (LV) wet weight, and (d) right ventricular (RV) wet weight. Mean ± SEM was used, and for statistical calculation, one-way ANOVA was conducted followed by a Tukey test, where *N* = 6. Statistical significance was considered at *P* < 0.05. Here, ns means *P* > 0.05, ^*∗*^means *P* ≤ 0.05, and ^*∗∗*^means *P* ≤ 0.01.

The kidney wet weight of the 2K1C rats increased significantly (*P* ≤ 0.01) compared to that of the control rats (Figure 2(c)). The *Crataeva nurvala* bark extract did not alter kidney wet weight compared to that in 2K1C rats. In comparison to the 2K1C group, the kidney wet weight of the 2K1C + Ram rats also did not change (*P* ≤ 0.01) (Figure 2(c)).

The 2K1C rats showed increased heart wet weight significantly (*P* > 0.05) (Figure 2(d)) compared to the control rats. The 2K1C rats also showed increased LV and RV wet weight of the heart compared to the control rats. However, the *Crataeva nurvala* bark extract did not alter the heart wet weight in 2K1C rats (Figure 2(d)).

### 4.3. Effects of the *Crataeva nurvala* Bark on AST, ALT, and ALP Levels in Plasma of 2K1C Rats

**Figure 3:**
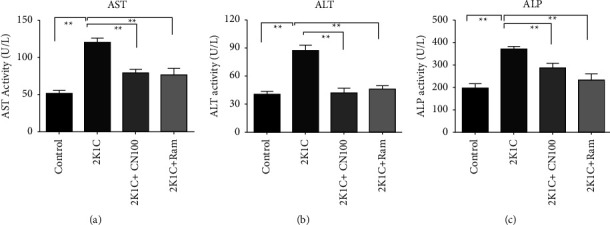
Effect of the *Crataeva nurvala* bark extract on ALT, AST, and ALP activities of 2K1C rats. (a) ALT, (b) AST, and (c) ALP. Mean ± SEM was used, and for statistical calculation, one-way ANOVA was conducted followed by a Tukey test, where *N* = 6. Statistical significance was considered at *P* < 0.05. Here, ns means *P* > 0.05, ^*∗*^means *P* ≤ 0.05, and ^*∗∗*^means *P* ≤ 0.01.

The 2K1C rats showed significantly higher activities of ALT and AST (*P* ≤ 0.01) (Figures 3(a) and 3(b)) in plasma compared to the control rats. The *Crataeva nurvala* bark extract helped normalize their high ALT and AST enzyme activity significantly (*P* ≤ 0.01) compared to 2K1C rats (Figures 3(a) and 3(b)). Similarly, ALP activity was shown to be increased significantly (*P* ≤ 0.01) in 2K1C rats compared to that in the control rats, and these increased ALP activities were significantly (*P* ≤ 0.01) inhibited by the *Crataeva nurvala* bark extract and ramipril treatment (Figure 3(c)).

### 4.4. Effects of the *Crataeva nurvala* Bark on Uric Acid and Creatinine Concentration in Serum of 2K1C Rats

**Figure 4:**
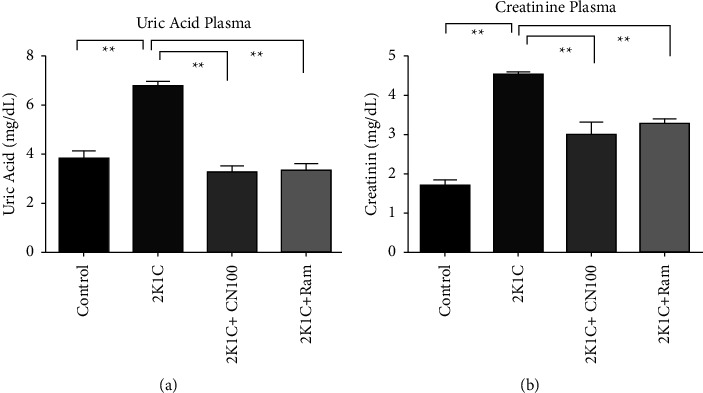
Effect of the *Crataeva nurvala* bark extract on uric acid and creatinine levels in plasma of 2K1C rats. (a) Uric acid plasma and (b) creatinine plasma. Mean ± SEM (standard error mean) was used, and for statistical calculation, one-way ANOVA was conducted followed by a Tukey test, where *N* = 6. Statistical significance was considered at *P* < 0.05. Here, ns means *P* > 0.05, ^*∗*^means *P* ≤ 0.05, and ^*∗∗*^means *P* ≤ 0.01.

Two essential markers of renal functions are uric acid and creatinine. The 2K1C rats showed significantly higher uric acid levels (*P* ≤ 0.01) than the control rats. *Crataeva nurvala* bark treatment considerably (*P* ≤ 0.01) lowered the uric acid level in plasma of 2K1C rats (Figure 4(a)). Creatinine levels in the plasma of 2K1C rats were significantly increased (*P* ≤ 0.01) compared to those in the control rats, which were lowered (*P* ≤ 0.01) by *Crataeva nurvala* bark treatment (Figure 4(b)). Moreover, uric acid and creatinine levels were also considerably lowered by ramipril treatment (Figure 4).

### 4.5. Effects of the *Crataeva nurvala* Bark on Oxidative Stress Markers in Plasma and Kidney and Heart Tissues of 2K1C Rats

**Figure 5:**
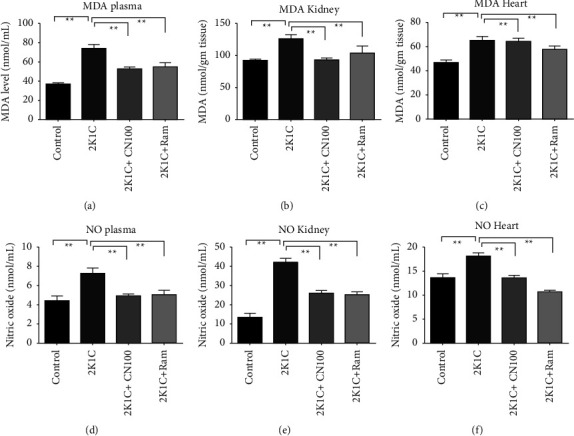
Effect of the *Crataeva nurvala* bark ethanol extract on oxidative parameters in the plasma, kidney, and heart of 2K1C rats. (a) MDA plasma, (b) MDA kidney, (c) MDA heart, (d) NO plasma, (e) NO kidney, and (f) NO heart. Mean ± SEM was used, and for statistical calculation, one-way ANOVA was conducted followed by a Tukey test, where *N* = 6. Statistical significance was considered at *P* < 0.05. Here, ns means *P* > 0.05, ^*∗*^means *P* ≤ 0.05, and ^*∗∗*^means *P* ≤ 0.01.

The results of this study revealed that oxidative stress markers were higher in the plasma, heart, and kidneys of 2K1C rats. Malondialdehyde (MDA), a lipid peroxidation product, is the most visible oxidative stress parameter. When compared to control rats, 2K1C rats showed significantly (*P* ≤ 0.01) higher MDA levels in plasma, heart, and kidney homogenates, indicating increased lipid peroxidation. *Crataeva nurvala* bark extract treatment significantly reduced or normalized the elevated MDA formation in plasma, kidney, and heart tissue homogenates (*P* ≤ 0.01) (Figures 5(a)–5(c)) in 2K1C rats.

Another oxidative stress parameter NO in the plasma, kidney, and heart was significantly (*P* ≤ 0.01) increased in 2K1C rats compared to that in the control rats. Rats treated with the *Crataeva nurvala* bark extract at 100 mg/kg significantly (*P* < 0.01) reduced NO in the plasma, kidney, and heart of 2K1C rats (Figures 5(d)–5(f)). Rats given ramipril also significantly lowered (*P* ≤ 0.01) NO concentrations in all samples of 2K1C rats.

**Figure 6:**
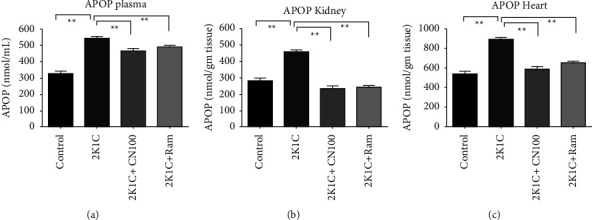
Effect of the *Crataeva nurvala* bark extract on the APOP level in the plasma (a), kidney (b), and heart (c) of 2K1C rats. Mean ± SEM was used, and for statistical calculation, one-way ANOVA was conducted followed by a Tukey test, where *N* = 6. Statistical significance was considered at *P* < 0.05. Here, ns means *P* > 0.05, ^*∗*^means *P* ≤ 0.05, and ^*∗∗*^means *P* ≤ 0.01.

Another parameter in this line of work is an advanced protein oxidation product (APOP), which is an influential oxidative stress marker in tissues that is yielded when free radicals react with tissue proteins. APOP concentrations were significantly (*P* ≤ 0.01) higher in the plasma, heart, and kidney tissue homogenates of 2K1C rats. *Crataeva nurvala* bark extract treatment significantly (*P* ≤ 0.01) lowered the APOP concentrations in 2K1C rats (Figures 6(a)–6(c)).

### 4.6. Effect of the *Crataeva nurvala* Bark Extract on Antioxidant Enzyme Activity in Plasma, Kidney, and Heart Tissue Homogenates of 2K1C Rats

**Figure 7:**
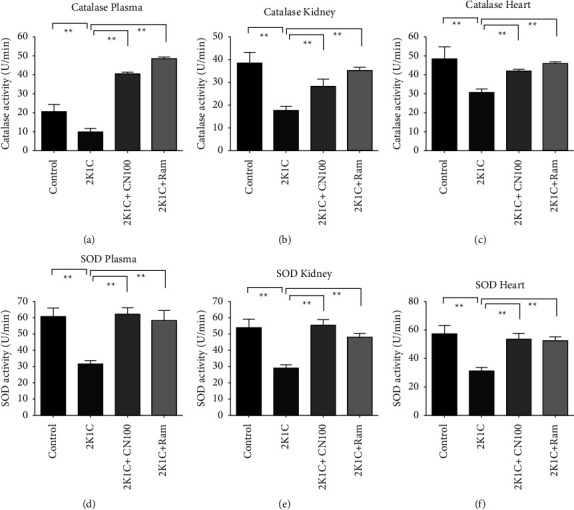
Effect of the *Crataeva nurvala* bark extract on antioxidant enzyme activity in the plasma, kidney, and heart of 2K1C rats. (a) Catalase plasma, (b) catalase kidney, (c) catalase heart, (d) SOD plasma, (e) SOD kidney, and (f) SOD heart. Mean ± SEM was used, and for statistical calculation, one-way ANOVA was conducted followed by a Tukey test, where *N* = 6. Statistical significance was considered at *P* < 0.05. Here, ns means *P* > 0.05, ^*∗*^means *P* ≤ 0.05, and ^*∗∗*^means *P* ≤ 0.01.

The antioxidant enzyme activities were significantly affected in 2K1C rats compared to those in the control rats. The catalase and SOD enzymes activities were found to be significantly (*P* ≤ 0.01) lowered in the plasma and tissue homogenates of 2K1C rats compared to those in the control rats (Figures 7(a)–7(f)). This study found that the *Crataeva nurvala* bark extract increased catalase activities in the plasma and tissue homogenates of 2K1C rats compared to control rats. Furthermore, the ramipril treatment in 2K1C rats significantly restored the (*P* ≤ 0.01) enzymes activities in the plasma, kidney, and heart compared to the control rats.

### 4.7. Effect of the *Crataeva nurvala* Bark Extract on CK-MB Activity in Plasma and MPO Activity in the Kidney and Heart of 2K1C Rats

**Figure 8:**
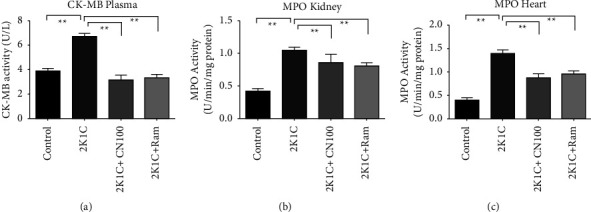
Effect of the *Crataeva nurvala* bark extract on CK-MB activity in the heart and MPO activity in the kidney and heart of 2K1C rats. (a) CK-MB heart, (b) MPO kidney, and (c) MPO heart. Mean ± SEM was used, and for statistical calculation, one-way ANOVA was conducted followed by a Tukey test, where *N* = 6. Statistical significance was considered at *P* < 0.05. Here, ns means *P* > 0.05, ^*∗*^means *P* ≤ 0.05, and ^*∗∗*^means *P* ≤ 0.01.

Creatinine kinase-muscle/brain (CK-MB) is a particular marker of heart function that is uniquely prevalent in the myocardium and released in circulation following myocardial injury or infarction. The results of this investigation showed that plasma CK-MB activities were significantly increased (*P* ≤ 0.01) in 2K1C rats compared to those in the control rats. Treatment with the *Crataeva nurvala* bark extract ameliorated elevated plasma CK-MB activities in 2K1C rats. Ramipril treatment also significantly (*P* ≤ 0.01) decreased the CK-MB activity in 2K1C rats (Figure 8(a)).

Again, MPO activities in the kidney and heart were significantly (*P* ≤ 0.01) higher in 2K1C rats than those in the control rats. The *Crataeva nurvala* bark extract ameliorated MPO activities in the kidney and heart significantly (*P* ≤ 0.01) in 2K1C rats (Figures 8(b) and 8(c)). Ramipril treatment also decreased (*P* ≤ 0.01) MPO activities in the kidney and heart of 2K1C rats.

### 4.8. Effect of the *Crataeva nurvala* Bark Extract on the Gene Expression of Inflammatory Mediators in the Kidney of 2K1C Rats

**Figure 9:**
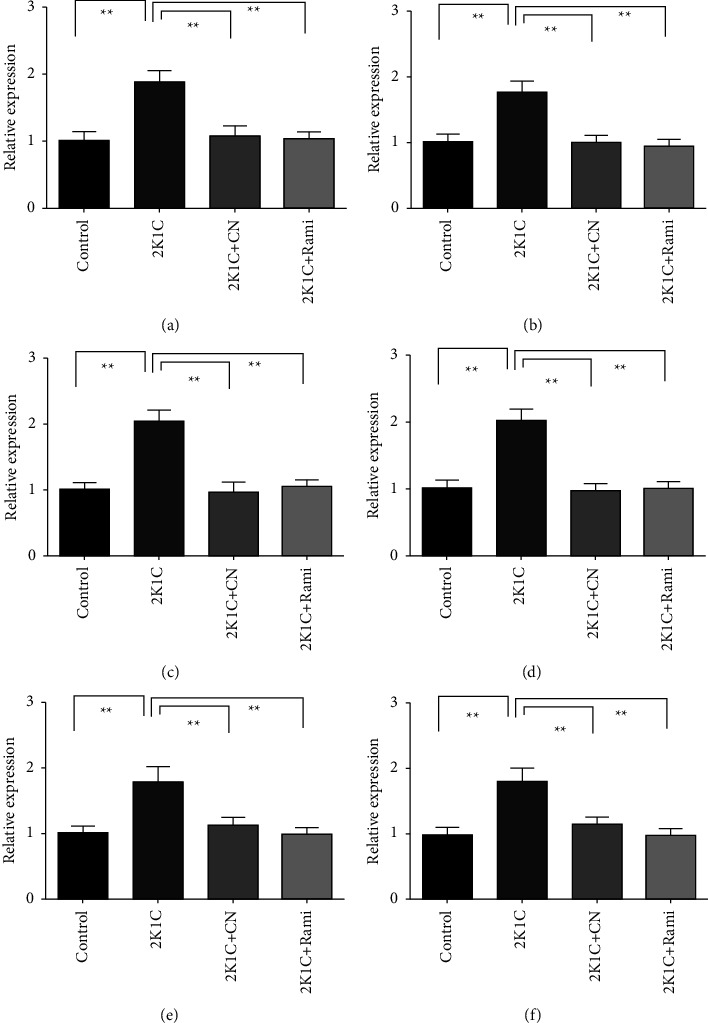
Effect of the *Crataeva nurvala* bark ethanol extract on inflammatory gene expression in the kidney of 2K1C rats. (a) IL-1, (b) IL-6, (c) TNF-*α*, (d) TGF-*β*1, (e) iNOS, and (f) NF-*к*B. Mean ± SEM was used, and for statistical calculation, one-way ANOVA was conducted followed by a Tukey test, where *N* = 6. Statistical significance was considered at *P* < 0.05. Here, ns means *P* > 0.05, ^*∗*^means *P* ≤ 0.05, and ^*∗∗*^means *P* ≤ 0.01.

To gain a better understanding of the effect of the *Crataeva nurvala* bark on the transcript levels related to inflammation and fibrosis, the gene expression of inducible nitric oxide synthase (iNOS), inflammatory cytokines including interleukin-1 (IL-1) and interleukin-6, (IL-6), tumor necrosis factor alpha (TNF-*α*), transforming growth factor beta-1 (TGF-*β*1), and nuclear factor kappa-light-chain-enhancer of activated B cells (NF-*κ*B) were analyzed (Figure 9). This investigation found that that the transcript levels of iNOS, IL-1, IL-6, TNF-*α*, TGF-*β*1, and NF-*κ*B significantly (*P*  < 0.05) increased in the renal tissues of 2K1C rats. Treatment with the *Crataeva nurvala* bark extract significantly (*P*  < 0.05) subdued the mRNA levels of these enzymes, cytokines, and transcription factors. This ameliorating action of the *Crataeva nurvala* bark extract is also comparable to the ramipril treatment which also lowered the inflammatory cytokine expression in the kidneys of 2K1C rats (Figure 9).

### 4.9. Effect of the *Crataeva nurvala* Bark Extract on the Gene Expression of Antioxidant Mediators in the Kidney of 2K1C Rats

**Figure 10:**
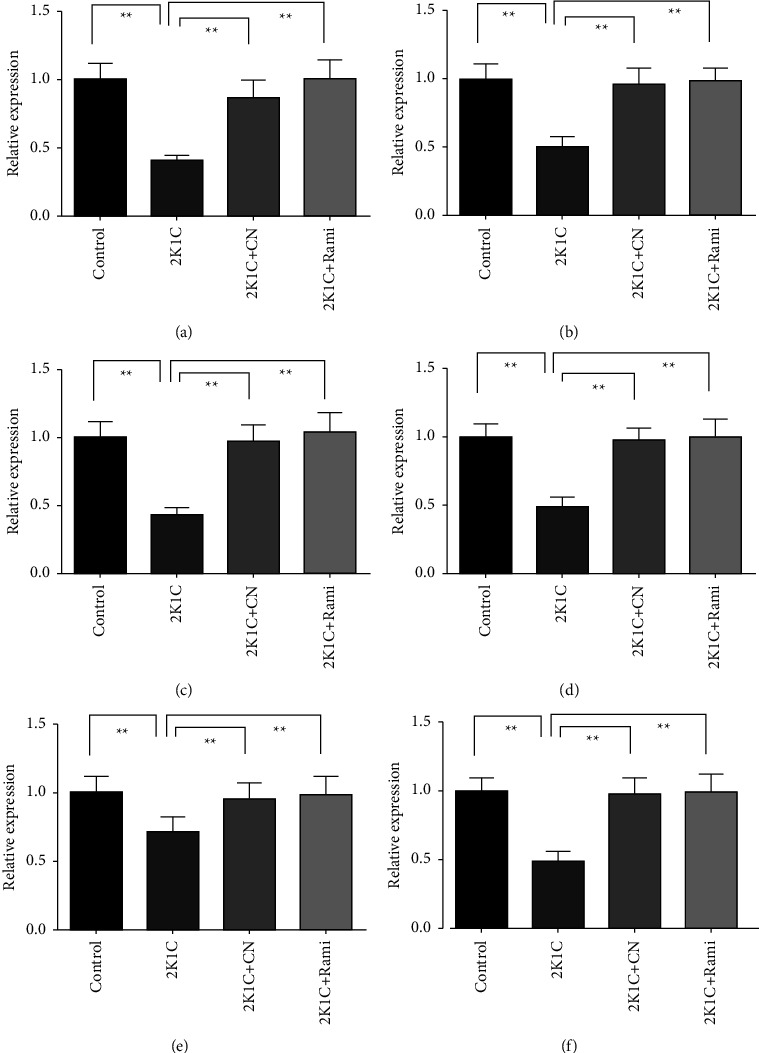
Effect of the *Crataeva nurvala* bark extract on antioxidant gene expression in the kidney of 2K1C rats. (a) Nrf-2, (b) HO-1, (c) HO-2, (d) SOD, (e) catalase, and (f) GPx. Mean ± SEM was used, and for statistical calculation, one-way ANOVA was conducted followed by a Tukey test, where *N* = 6. Statistical significance was considered at *P* < 0.05. Here, ns means *P* > 0.05, ^*∗*^means *P* ≤ 0.05, and ^*∗∗*^means *P* ≤ 0.01.

The nuclear factor erythroid 2-related factor 2 (Nrf-2) is a multifunctional cytoprotective protein that regulates the expression of antioxidant and anti-inflammatory genes. The expression level of Nrf-2 was compromised in 2K1C rats compared to that in control rats. The Nrf-2 transcript level was restored by the *Crataeva nurvala* bark extract significantly (*P* ≤ 0.01) (Figure 10). As a result, the inducible and constitutive isoforms of heme oxygenases (HO-1, and HO-2, respectively) were significantly (*P* ≤ 0.01) restored in 2K1C rats (Figure 10). As a result, the inducible and constitutive isoforms of hemeoxygenases (HO-1, and HO-2, respectively) were significantly (*P* ≤ 0.01) restored in 2K1C rats by Crataeva nurvala bark extract treatment (Figure 10). Ramipril treatment also increased these genes (i.e., Nrf-2, HO-1, and HO-2 genes) significantly (*P* ≤ 0.01) in 2K1C rats. Furthermore, gene expressions of antioxidant enzymes such as glutathione peroxidase (GPx), SOD, and catalase were also elevated due to the *Crataeva nurvala* bark extract or ramipril treatment (Figures 10(d)–10(f)).

### 4.10. Effect of the *Crataeva nurvala* Bark Extract on Tissue Histology of the Kidney and Heart of 2K1C Rats

**Figure 11:**
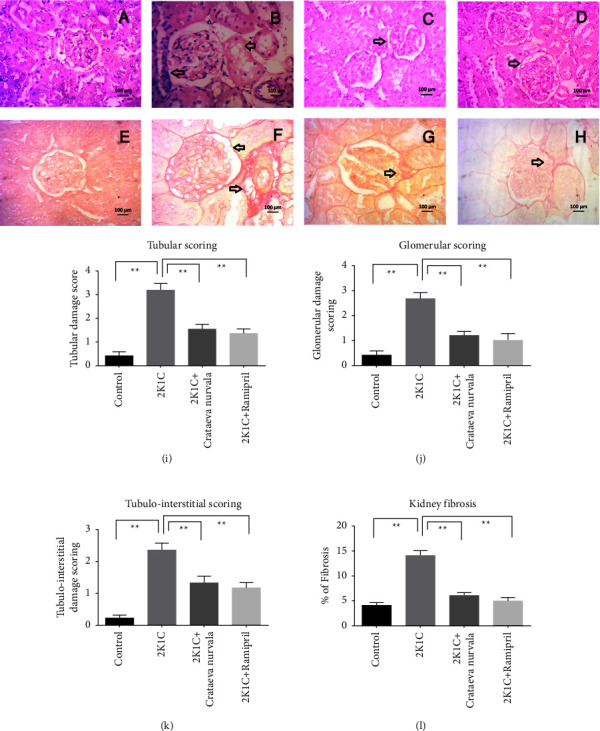
Effect of the *Crataeva nurvala* bark extract on the kidney and fibrosis in 2K1C rats. (a, e) Control, (b, f) 2K1C, (c, g) 2K1C + *Crataeva nurvala*, and (d, h) 2K1C + ramipril. Magnification 40x. (i) Tubular scoring, (j) glomerular scoring, (k) tubulointerstitial scoring, and (l) % of kidney fibrosis. Mean ± SEM was used, and for statistical calculation, one-way ANOVA was conducted followed by a Tukey test, where *N* = 6. Statistical significance was considered at *P* < 0.05. Here, ns means *P* > 0.05, ^*∗*^means *P* ≤ 0.05, and ^*∗∗*^means *P* ≤ 0.01.

A representative micrograph showed kidney histomorphology in control rats (A) and rats that underwent 2K1C surgery (B), 2K1C + *Cratavae nurvala* (C), or 2K1C + ramipril (D) using H&E staining. No changes were seen in the kidney section of control rats (Figure 11(a)). However, marked expansion and hypercellularity of the interstitial area were noted in 2K1C rats (Figure 11(b)), along with increased amounts of collagen-like material (Figure 11(f)). 2K1C rats also showed vacuolar degeneration of epithelial lining renal tubules, congestion of renal blood vessels and glomerular tufts, and focal necrosis of epithelial lining of renal tubules (Figure 11(b)). Treatment with the *Cratavae nurvala* bark extract reduced the degeneration of the kidney structure in 2K1C rats (Figure 11(c)) and prevented fibrosis development (Figure 11(g)). A similar protective effect was also observed in the treatment with ramipril in 2K1C rats (Figures 11(d) and 11(h)). The histological changes in the kidneys are semiquantitatively analyzed and presented in Figures 11(i)–11(l). % of fibrosis significantly increased in 2K1C rats compared to that in control rats, which was significantly normalized by *Crataeva nurvala* bark extract treatment (Figure 11(l)).

**Figure 12:**
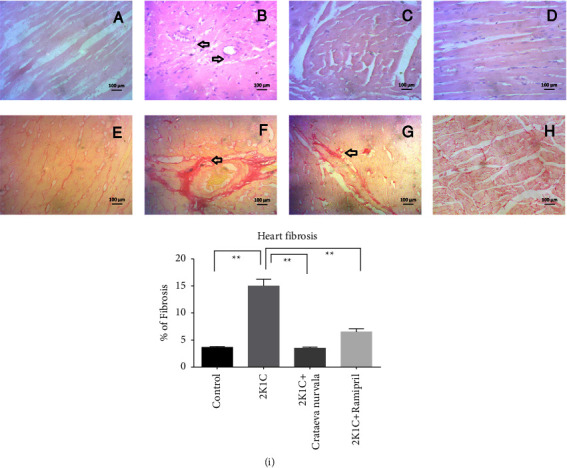
Effect of the *Crataeva nurvala* bark extract on the heart and fibrosis in 2K1C rats. (a, e) Control, (b, f) 2K1C, (c, g) 2K1C + *Crataeva nurvala*, (d, h)-2K1C + ramipril, and (i) % of heart fibrosis. Mean ± SEM was used, and for statistical calculation, one-way ANOVA was conducted followed by a Tukey test. Statistical significance was considered at *P* < 0.05. Here, ns means *P* > 0.05, ^*∗*^means *P* ≤ 0.05, and ^*∗∗*^means *P* ≤ 0.01.

Histopathological features of the left ventricle myocardium stained with H&E and sirius red staining are illustrated in Figure 12. Control rats (Figure 12(a)) showed normal myocardium histology. The only obvious qualitative feature in 2K1C rats was the presence of diffuse fibrosis and myocardial hypertrophy compared to control rats (Figures 12(b) and 12(f)). Treatment with the *Cratavae nurvala* bark extract reduced the necrosis of the myocardium in 2K1C rats (Figure 12(c)) and prevented fibrosis development (Figure 12(g)). A similar protective effect was also observed in the treatment with ramipril in 2K1C rats (Figures 12(d) and 12(h)). % of fibrosis in the left ventricle of the heart in 2K1C rats is presented in Figure 12(e). 2K1C rats showed increased collagen deposition and fibrosis which were reduced by *Cratavae nurvala* bark extract treatment (Figure 12(e)).

## 5. Discussion

*Crataeva nurvala* is a valuable medicinal plant in Unani and Ayurveda systems. It contains several polyphenolic compounds which can protect the kidney and heart from oxidative stress in 2K1C rats. This study revealed that the bark of the *Crataeva nurvala* extract possesses catechin hydrate, catechol, (−) epicatechin, vanillic acid, rutin hydrate, rosmarinic acid, myricetin, and trans-cinnamic acid. The extract of *Crataeva nurvala* bark also prevented lipid peroxidation, restored antioxidant enzyme function, and modulated several genes involved in inflammation and oxidative stress in the kidney of 2K1C rats.

Tissue damage and cellular necrosis are caused by disruption of membrane lipid layers, which happens directly because of free radical interactions with lipids [39]. There are several sources and mechanisms for free radical production such as industrial toxin, chemical mediators, iNOS activity, NADPH activity, and abnormal mitochondrial function [40]. An increment in ROS production, NADPH activity, and mitochondrial function may be regulated by angiotensin II [41]. The antioxidant defense system may overwhelm because of over production of free radicals, especially ROS [42]. ROS may cause oxidation of intracellular and extracellular protein mainly cysteine residue of thiol side chains [43]. Complications of renal and cardiac systems are also related to lipid peroxidation [37, 44]. A previous report suggested that oxidative stress evidenced by increased lipid peroxidation, nitric oxide production, and reduced SOD and catalase activities are prevalent in the kidney and heart of 2K1C rats [28, 29]. This study revealed that renal and cardiac damages were prevented by the *Crataeva nurvala* bark extract by decreasing lipid peroxidation and nitric oxide production and restoring SOD and catalase activities. This result can be supported by a previous study that reported that the ethanol extract of *Crataeva nurvala* stem bark showed protection in cisplatin-induced nephrotoxicity and kidney dysfunction [45]. In another study, it was revealed that oxidative stress induced by N-methyl-N-nitrosourea and testosterone elevated the concentration of MDA, which was controlled by the bark extract of *Crataeva nurvala* [46].

A significant number of physiological functions in the human body depend on nitric oxide. Many different kinds of pathogenesis are also related to nitric oxide [47]. Nitrosative stress can damage tissue which is caused by generation of peroxynitrite (ONOO^−^) [48]. In this study, the NO level was found to be increased in 2K1C rats. The *Crataeva nurvala* extract normalized the NO level in plasma and tissues of 2K1C rats. This result is supported by the previous report that showed that *Crataeva nurvala* treatment lowered the NO level in lipopolysaccharide (LPS)-stimulated RAW 264.7 [20]. This study also revealed that the *Crataeva nurvala* extract prevented the iNOS mRNA expression and reduced inflammation by negative regulation of ERK in murine macrophages [20]. In this study, the AOPP level in plasma and tissues increased in 2K1C rats, which was decreased by *Crataeva nurvala* treatment. It can be stipulated that polyphenolic compounds due to their antioxidant activity may decrease the AOPP level in oxidative stress [28].

The reduction of oxidative stress may also depend on the restoration of antioxidant enzyme activities such as SOD and catalase. SOD mainly scavenges superoxide radicles to hydrogen peroxide (H_2_O_2_), and catalase converts H_2_O_2_ to hydroxyl radical and ultimately water [49]. Catalase and SOD activities were restored in 2K1C rats by the treatment of the *Crataeva nurvala* bark extract. A previous report also supports this finding that both catalase and SOD may be restored by *Crataeva nurvala* bark extract treatment [46]. Antioxidant enzymes are regulated by nuclear factor erythroid 2-related factor 2 (Nrf-2) which is a transcriptional factor activated against oxidative stress to stimulate tissue defense [50]. In this study, the kidney tissues showed the decreased expression in Nrf-2 and its downregulatory gene HO-1. *Crataeva nurvala* bark extract treatment restored the Nrf-2-HO1 axis and increased the SOD, catalase, and GPx expression in the kidney of 2K1C rats. This finding is similar to that of a previous report that showed that antioxidant therapy may prevent the progression of diabetic nephropathy partly by modulating the Nrf-2 pathway [51]. Uric acid and creatinine are another two specific markers associated with kidney dysfunction [52]. In this study, it was found that both uric acid and creatinine levels increased in 2K1C rats, which were decreased by the treatment of the *Crataeva nurvala* bark extract. In another study, it was confirmed that *Crataeva nurvala* decreased both uric acid and creatinine levels [25].

Chronic kidney dysfunction may also develop cardiac complications and cardiovascular diseases. The 2K1C rat model showed cardiac dysfunction because of the activation of the renin-angiotensin system. Angiotensin II activation caused the stimulation of transforming growth factor-beta (TGF-*β*) and vascular endothelial growth factors which cause cellular growth and hypertrophy in the kidney and heart. Angiotensin II activation may also stimulate nuclear factor kappa B (NF-*к*B) which causes fibrosis and inflammation [53]. One of the cardinal signs of cardiac damage is the release of CK-MB in plasma. In this study, CK-MB activities were found to be increased in 2K1C rats as a sign of cardiac damage. The *Crataeva nurvala* extract reduced the CK-MB activity in plasma of 2K1C rats and prevented cardiac damage due to oxidative stress. This response is also comparable to ramipril, which is an angiotensin-converting enzyme (ACE) inhibitor, treatment in 2K1C rats. Other reports also showed that the *Crataeva nurvala* bark extract possesses lupeol [54] which may exert cardiac protective activity against oxidative stress induced by cyclophosphamide [55].

Oxidative stress in the heart and kidneys also attracts inflammation and may develop fibrosis in 2K1C rats. A previous finding suggests that fibrosis is developed in the left ventricle of the heart because of the role of ROS and oxidative stress [56]. Cardiovascular damage and endothelial damage may be increased in chronic kidney patients [57]. Angiotensin II activates signaling pathways such as chemoattractants attract inflammatory cells mainly macrophages to injury sites [53]. As suggested in this study, the 2K1C rats showed increased MPO activity as a sign of inflammatory cell infiltration in both kidney and heart tissues which were neutralized by the *Crataeva nurvala* bark extract. Histological assessment in the kidney and heart showed the collagen deposition in 2K1C rats which is correlated with the inflammatory gene expression such as TNF-*α*, IL-6, and TGF-*β* found in the kidney. Inflammatory cytokines and TGF-*β* signaling are the primary player in the development of fibrosis in the kidneys [58]. Oxidative stress and the cytokine expression may work synergistically to develop fibrosis in tissues mainly in the kidneys and heart of 2K1C rats [28, 37]. The *Crataeva nurvala* bark extract significantly lowered the cytokine expression followed by the reduced collagen positive area in the kidneys and heart of 2K1C rats. This result is also comparable to the effect of ramipril treatment in 2K1C rats, which showed significant prevention in collagen deposition and fibrosis.

## 6. Conclusion

From this study, it can be assumed that the *Crataeva nurvala* bark extract reduced fibrosis, inflammation, cardiovascular complications, and renal dysfunction and improved antioxidant capability in 2K1C rats. Further investigation is warranted to establish the formulation with the *Crataeva nurvala* bark extract and its efficacy in the clinical setup.

## Figures and Tables

**Table 1:** The forward and reverse sequence of the primers applied in this experiment.

Name	Type	Sequence	Tm value (°C)	Aplicon	Accession no.
Catalase	Forward	5-ATTGCCGTCCGATTCTCC-3	60.1	155	AH004967.2
	Reverse	5-CCAGTTACCATCTTCAGTGTAG-3	59.6		

MnSOD	Forward	5-GCTCTAATCACGACCCACT-3	62.3	147	NM_017051.2
	Reverse	5-CATTCTCCCAGTTGATTACATTC-3	61.5		

GPx	Forward	5-CAGTTCGGACATCAGGAGAAT-3	61.3	139	S50336.1
	Reverse	5-AGAGCGGGTGAGCCTTCT-3	60.8		

Heme oxygenase-1	Forward	5-TGCTCGCATGAACACTCTG-3	60.1	123	NM 012580.2
	Reverse	5-TCCTCTGTCAGCAGTGCCT-3	59.3		

HO-2	Forward	5-CACCACTGCACTTTACTTCA-3	62.3	231	J05405.1
	Reverse	5-AGTGCTGGGGAGTTTTAGTG-3	61.5		

Nrf-2	Forward	5-CCC AGCACA TCC AGACAGAC-3	60.4	125	XM_213329
	Reverse	5-TATCCAGGGCAAGCGACT C-3	61.7		

IL-1	Forward	5-ATGCCTCGTGCTGTCTGACC-3	60.7	172	M98820
	Reverse	5-CCATCTTTAGAGACACGGGTT-3	60.8		

IL-6	Forward	5-AGCGATGATGCACTGTCAGA-3	61.3	168	M26744
	Reverse	5-GGTTTGCCGAGTAGACCTCA-3	58.9		

TGF-*β*1	Forward	5′-AAGAAGTCACCCGCGTGCTA-3′	59.2	187	NM_021578.2
	Reverse	5′-TGTGTGATGTCTTGTTTTGTC-3′	58.7		

iNOS	Forward	5′-TGGTCCAACCTGCAGGTCTTC-3′	60.1	213	NM_012611.3
	Reverse	5′-CAGTAATGGCCGCTGATGTTG-3′	59.8		

TNF*α*	Forward	5′-ATGTGGAACTGGCAGAGGAG-3′	61.2	169	L00981.1
	Reverse	5′-CCACGAGCAGGAATGAAGAG-3′	58.9		

NF-*κ*B (*P*65)	Forward	5′-TGTGAAGAAGCGAGACGGAG-3′	59.6	185	NM_001276711.1
	Reverse	5′-GGCACGGTTATAAATCGGATG-3′	60.8		

*β*-Actin	Forward	5′-GCGAGAAGATGACCCAGATC-3′	58.4	194	V01217.1
	Reverse	5′-GGATAGCACAGCCTGGATAG-3′	59.4		

**Table 2:** Compounds of *Crataeva nurvala* bark ethanol extract.

Name of standard compounds of the *Crataeva nurvala* (CN) bark ethanol extract	*Crataeva nurvala* (mg/100 g dry extract)
Catechin hydrate	5.28 ± 0.17
Catechol	1.25 ± 0.11
(−) epicatechin	10.88 ± 0.45
Vanillic acid	1.68 ± 0.05
Rutin hydrate	15.85 ± 0.54
Rosmarinic acid	18.89 ± 0.85
Myricetin	4.10 ± 0.19
Trans-cinnamic acid	4.85 ± 0.23

## Data Availability

The data used to support the findings of this study are included within the article.

## Abbreviations

ANOVA: One-way analysis of variance
AST: Aspartate aminotransferase
ALT: Alanine transaminase
ALP: Alkaline phosphatase
APOP: Advanced protein oxidation product
CAT: Catalase
CK: Creatine kinase
CK-MB: Creatine kinase-MB
GPx: Glutathione peroxidase
GSH: Reduced glutathione
H&E: Hematoxylin and eosin stain
H_2_O_2_: Hydrogen peroxide
IACUC: Institutional Animal Care and Use Committee
IL: Interleukins
iNOS: Nitric oxide synthases
MDA: Malondialdehyde or 1,1,3,3-tetramethoxypropane
MPO: Myloperoxidase
NO: Nitric oxide
Nrf-2: Nuclear factor erythroid 2-related factor 2
NF-*к*B: Nuclear factor kappa-light-chain-enhancer of activated B cells
ROS: Reactive oxygen species
TNF-*α*: Tumor necrosis factor alpha
TGF-*β*: Transforming growth factor-*β*
SEM: Standard error of the mean
SOD: Superoxide dismutase
TBA: Thiobarbituric acid
TCA: Trichloroacetic acid.
